# Highlighting common pitfalls to avoid when writing the medical education manuscript

**DOI:** 10.15694/mep.2017.000094

**Published:** 2017-06-06

**Authors:** B. Lee Ligon, Teri L. Turner, Satid Thammasitboon

**Affiliations:** 1 Baylor College of Medicine

**Keywords:** Scholarship, Plagiarism, Publication, Discourse

## Abstract

This article was migrated. The article was marked as recommended.

Medical educators have an unprecedented opportunity to advance the field through dissemination of their work in academic publications. Also, their advancement may depend heavily on the number of publications. However, writing is, for many medical educators, a daunting task. Fortunately, authors have provided valuable articles and tips on the “how-to” of writing, and faculty development workshops have provided venues during which one can write a basic paper. These all are valuable, but they do not cover some of the unforeseen potential problems associated with publishing. Hence, we offer common pitfalls that the unsuspecting author will want to avoid, grouped into four categories--ethics of publishing, aims of discourse, setting boundaries, and accountability--in a reflective framework that most educators will recognize. These highlights should better equip novice medical educators, provide insights for experienced medical education mentors, and enhance the likelihood that the scholarly work will be published.

## Introduction

With the field of medical education research being a relatively newer career path, medical educators have an unprecedented opportunity to advance the field through dissemination of their work in academic publications. Further, a medical educator’s advancement may depend heavily on the number of articles published ( Cable, Boyer, Colbert, Boyer 2013 Malani & Lypson 2012; Simpson, McLaughlin, Schiedermayer 2000), requiring that the medical educator *write* up the scholarly work and submit it, a daunting task for some individuals ( Boice 1989; Keyes 1995; Malani & Lypson 2012; Steinert, McLeod, Liben, Snell 2008; Sullivan 2013), especially those who received little or no education in writing during their years of training ( Sullivan 2013; Singh & Mayer 2014). Fortunately, authors have succinctly addressed the “how-to” of writing manuscripts in articles and shorter correspondence (Azer, Dupras, Azer 2014; Cook 2015), and faculty development workshops have provided venues during which the novice can write a basic paper. These all are valuable contributions, but they do not cover some of the unforeseen potential problems associated with publishing. Hence, we address the pitfalls that the unsuspecting author will want to avoid, as they serve to hinder or even prevent the publication of one’s scholarly work. They are grouped into four categories-ethics of publishing, aims of discourse, setting boundaries, and accountability-in a reflective framework that most educators will recognize. These highlights should better equip novice medical educators, provide insights for experienced medical education mentors, and enhance the likelihood that the scholarly work will be published.

## Ethics of Publishing

Scholars are aware of the ethical standards of their own disciplines, but many are unaware that the publishing field has its own ethical standards ( Graf 2006), and/or that ignoring or violating them can have dire consequences. The Committee of Publications Ethics (COPE) (publicationethics.org) addresses these areas in detail, so we will focus on three of the primary ethical pitfalls we have encountered with authors (including senior faculty), namely falsified literature research, questionable authorship, and plagiarism. We urge the reader to go to the COPE website for detailed information on other topics.

### Highlight 1: “What’s wrong with using someone else’s citations?” (Do your own research!)

Performing a literature search has become increasingly efficient with the development of the internet, and usually authors can access most of the literature they need through electronic means. Nonetheless, a tendency to take a short cut that can be disastrous continues, namely using data and information in a published article(s) and supplying the accompanying citation without checking the primary source. One such example is the application of Dale’s “Cone of Learning,” used by medical educators as a theoretical basis for studies (“. . . the successful applications are based on theories and principles of the learning process developed by two pioneers in the field, Robert Gagne and Edgar Dale . . . . The extensive work of Dale as formulated in his Cone of Experience relates the effectiveness to the efficiency of education activities” [ Sprawls 2008)]) and for statistical analysis (“The goal of this study will be to examine how Edger [sic] Dale’s Cone of Experience is employed to positively impact student learning in a foundational leadership course . . . . This approach will focus on a self-assessment survey to gather in-depth understanding of learning and the reasons that student learning occurred as a result application of Edger [sic] Dale’s Cone of Experience” [ Davis 2014]). Apparently unbeknownst to the authors who use this “cone” is that the “Dale’s Cone” as such does not even exist. Edgar Dale did not base his study on scientific research, included no numbers/percentages, and warned against taking it too seriously ( Dale 1954; Lalley & Miller 2007; Thalheimer 2006). The numbers and percentages were added later by D. G. Treichler, a Mobil Oil Company employee, who published it in a non-scholarly article in a film/audio magazine in 1967 ( Lalley & Miller 2007; Dwyer 2010). The point is: *do your homework.* It is incumbent upon the author to access *original* articles. Doing so is a matter of intellectual integrity - as well as a safeguard against misquoting.

### Highlight 2: “Does my mentoree qualify as an author?” (Be aware of criteria for authentic authorship.)

Identifying co-authors is a challenge, especially when one is asked (or required) to include individuals who have had only a cursory association with the study and/or the manuscript, and the medical educator may have difficulty determining who qualifies. You can avoid this pitfall by accessing the requirements established by COPE and other organizations (e.g., ICMJE, International Committee of Medical Journal Editors; http://www.icmje.org); also, many institutions now published these requirements and cite COPE. The qualifications, summarized, are: 1) made substantial contributions to the conception, design, and performance of the research or the acquisition of data and its analysis and interpretation; 2) participated in drafting and/or revising the article; 3) gave approval of the final draft; and 4) able to take public responsibility for appropriate portions of the content. COPE also has published a “discussion document” regarding authorship that should be helpful when questions arise (https://publicationethics.org/files/Authorship_DiscussionDocument.pdf) and an article on handling disputes about authorship ( Albert & Wager 2003).

### Highlight 3: “Yikes! I’ve seen this before!” (Know what constitutes plagiarism and vigorously avoid it!)

Although this ethical misstep is defined and taught in courses beginning in elementary school and emphasized through college, it continues to plague the publishing field, as well as the academic community. Indeed, explaining how to avoid plagiarism in scientific writing appears to be an ongoing challenge ( Fisher & Partin 2014). Plagiarism is, simply put, the use of someone else’s written work without giving proper credit; it is academic theft. Even short phrases lifted from another person’s article but not cited constitute plagiarism, as does paraphrasing that does not acknowledge the source. Because plagiarism has become so prevalent and is so easily, and often unintentionally, committed, most of the major publishing houses now use electronic programs such as CrossCheck ^TM^ as a means to detect plagiarism (http://www.ithenticate.com/products/crosscheck).

The repercussions of plagiarism can be serious and include public exposure and disgrace (COPE; Masic 2012; Chalmers 2006; Vessal & Habibzadeh 2007), as well as the author’s future articles being blocked by publishing houses.

A form of plagiarism that often is unintentional is “self-plagiarism” or “text recycling.” It involves using ideas from the author’s previously published article without citing that article. It is considered by some to be unethical ( Bruton 2014) and may result in copyright infringement by the new publisher, with accompanying legal issues ( Dellavalle, Banks, Ellis 2007; Rosing & Cury 2013). Although the concept remains controversial ( Bretag & Mahmud 2009; Andreescu 2013; Joob & Wiwanitkit 2016), we recommend erring on the side of caution: provide citations for everything you use from another source that is not general information.

## Aims of Discourse

Many years ago, James Kinneavy published a theory of discourse, based on the earlier work of Piaget on childhood development ( Kinneavy 1969). Basically, he grouped discourse into three areas or aims: expressive (the author is focused on self), referential (the focus is on information), and persuasive (the focus is on convincing the audience). The medical educator’s concerns are the latter two, respectively: describing his/her scholarly work and convincing the reader of its value. Three key matters in any persuasive discourse (with application to publishing) are: 1) identify the appropriate audience (the most appropriate journal); 2) persuade or “move” the audience by crafting a succinct rhetorical statement (state *why* the scholarly work is valuable); and 3) know when the support is adequate (know *when* the manuscript is ready to submit).

### Highlight 4: “Who is interested in or would benefit from my scholarly work?” (Select the appropriate audience.)

Three cautions accompany the identification of the most appropriate journal, and each is itself a pitfall: 1) starting “too high”: namely, submitting to a journal with a very high impact factor, as these journals receive thousands of submissions and are very competitive, so your research must be exceptional; 2) starting “too low”: contrariwise, submitting to a low-impact-factor journal may get easy acceptance but not the long-term prestige or recognition that your work deserves; and 3) using “predator journals.” A good source for identifying an appropriate journal is JANE, the Journal/Author/Name Estimator (http:www.biosemantics.org/jane/), an online means to search journals for authors, types of articles, and other options. We also recommend Scimago Journal & Country Rank (SJR) (scimagojr.com/journalrank.php), which sorts journals according H-index, total citations, and other data. With the increased launching of publications, one may have difficulty distinguishing between a predator journal and a legitimate open-access journal. After recognizing this problem, Jeffrey Beall, an academic librarian, began tracking the practice and then coined the term *predator publishers*, defining them is “those that unprofessionally abuse the author-pays publishing model for their own profit”(Beall 2012). In addition to tracking and identifying these journals, he provides some warning signs of questionable publishers. A recent initiative is the Think. Check. Submit (http://thinkchecksubmit.org), which was launched in 2015 and provides a “checklist” for the author to use to assess the journal’s legitimacy.

### Highlight 5: “So What? Why is your scholarly work important?” (Craft a rhetorical statement that articulates the value of your work.)

Many authors assume that if they simply *describe* their scholarly work, the reader will immediately recognize its value. Unfortunately, this is a dangerous way to proceed. You don’t want the reviewers or readers to look at your manuscript, scratch their heads, and ask, “So what?” In different portions, you will need to take a persuasive stance, articulating what value the scholarly work has and, at the end, stating what it means for the future. The term *rhetoric* has gotten a bad rap in recent years, but authentic, classical rhetoric is the art of *persuasion*, not trickery or deception. In this context, you will need to “persuade” the reader that your scholarly work is useful ( Overington 1977; Weimer 1977), by forming a rhetorical statement. One way to craft it is simply to answer succinctly one of these questions: What relevance does this scholarly work have for medical education? Why is this work important to me and other medical educators? What might these findings have for the future of medical education?

### Highlight 6: “Is my manuscript ready to send?” (Recognize if you are rushing to publication or delaying the process unnecessarily.)

With the pressure on many academicians to publish, a distinct pitfall has been to rush a document to submission, if only to demonstrate that one has publications “submitted.” Rather than being an advantage for the author, however, premature submission merely prolongs the process and can, in fact, eliminate the manuscript from being considered by a viable journal that rejects it on the basis of its insufficiencies. Several criteria should be met before the manuscript is considered ready to submit: 1) adds to the body of knowledge; 2) questions or refutes findings previously published; or 3) takes scholarly work to the next level, academically and /or logically. Once your manuscript has met these criteria and the co-authors have signed-off on it, then send it off as quickly as possible. The flip-side to premature submission is delayed submission, during which time, someone else may submit an article that “scoops” your scholarly work, or it may simply become outdated.

## Boundaries

The concept of setting boundaries with regard to the vernacular became a household phenomenon some years ago after the publication of the *New York Times* bestseller by Drs. Henry Cloud and John Townsend, entitled simply *Boundaries* ( Cloud & Townsend 1969). The subtitle was “when to say yes, how to say no, to take control of your life.” The concept of protecting oneself applies to the challenge of writing, as it does to other areas of our lives. The essence of setting boundaries is to protect ourselves and our time, for our own well-being. We will look at three areas of boundaries related to writing: time, space, and interruptions or distractions.

### Highlight 7: “No one understands that I need time to get this manuscript written!” (Protect the time you have designated for writing.)

For some reason, people often seem to think that reading and writing are invitations to be interrupted. We will address later the misconception authors have about making time to write, but *protecting* time for writing is as important as *making* time. One means is to carve out time on your calendar, which provides you with the answer, should someone want that time slot, that you already have something planned and on your calendar. Many people will have far more respect for your “calendar” than your actual time! Other tactics, such as shutting the door to your study or office, perhaps posting a friendly do-not-disturb sign, have been successful for some writers. It will be up to you to make sure your time is respected.

### Highlight 8: “I just can’t write in this office/house/area/etc.!” (Find a place where you can write -- uninterrupted.)

People who teach concepts of writing are fully familiar with a phenomenon that the medical educator may not be aware: the best *place* to write depends upon the individual’s personality, preferences, priorities, and other matters. Where I write well may not work for you..and vice versa. If you find yourself stumped with writing, take your materials to another setting, be it a library, a coffee shop, a picnic table under a tree - whatever works for *you*! If you need a very quiet setting, the library is an option; if, contrariwise, you need some peripheral “noise,” a coffee shop or restaurant might work better. Just make sure that you also establish a “space boundary” that says you don’t want to be interrupted (piles of books in front of you help!).

### Highlight 9: “Oops, there goes my phone again.” (Silence all mobile devices, if possible.)

A great distraction today is the incessant “notifications” of mobile devices. They can lead to fragmented work experiences, with a variety of results ( Mark, Gonzalez, Harris 2005). One study has shown that even brief and undemanding pop-up notifications may affect the worker’s efficiency, especially considering the frequency with which they occur ( Hodgetts & Jones 2007). Another study has shown that the typical office worker has only 11 minutes between interruptions and that 25 minutes is required to return to the original task after being interrupted ( Sullivan & Thompson 2013). Researchers who looked at various types of interruptions concluded that users spend far more time responding to alerts than they realize and are “unaware of the amount of time they end up spending on the alerting application, on other tasks they invoke as a result of responding to the alert, and on browsing through other peripheral applications before resuming the suspended task” ( Iqbal & Horvitz 2007). The solution: silence all devices so you don’t lose the precious time you have designated for writing!

## Accountability

The term *accountability* has numerous nuances in today’s world - we speak of holding people accountable or responsible, and philosophical debate continues about the definition and basis for accountability. The Psychology Dictionary defines it as “the degree to which someone is responsible for actions, decisions, and more” ( http://psychologydictionary.org/accountability/). In the context of writing, we use *accountability* to refer to the responsibilities we have to ourselves and to the other authors with regard to doing our research, writing, analyzing, and meeting deadlines. In the course of writing a multiple-author manuscript, mutual accountability is essential to the success of the collaborative process.

### Highlight 10: “I’m going to write that manuscript just as soon as . . . .” (Exercise discipline over procrastination.)

Writing often is such a challenge that putting if off - procrastinating - becomes almost an art form. The three excuses we have encountered most frequently relate to time, misconceptions of what is involved, and the myth that one “cannot write.” Each of these excuses is based on some perceived truth and requires more extensive inquiry than can be addressed herein, but we will note the misconceptions related to each.

The first misnomer is, “I just don’t have *time* to write.” Certainly, the workplace and family demands on one’s time are extensive, and many people must commute long distances to work, adding to their constraints on time. Nonetheless, one can, and often *must*, make time. Just as a pianist must be highly disciplined to practice or a jogger to run, for example, the writer must exercise discipline and set aside time. It may require serious re-adjustment of your schedule, but it can be done!

A second response has been related to the idea that “I just don’t feel inspired.” The reality is that writing in the fields of science, medicine, or education does not require inspiration - it requires discipline. It requires collecting the necessary materials and sitting down to write. An anonymous source has defined it this way: “Inspiration is the act of drawing up a chair to the writing desk.”

Another pitfall is believing the myth that “I simply can’t write.” You *can* write! Writing is a skill that can be gained and maintained. Good writing does not just happen - it requires numerous revisions (Hemingway wrote 47 endings to *A Farewell to Arms*!). Many people mistakenly think that their writing is poor because no one ever told them that a first draft is just that: a *first* draft! It is sometimes called a “rough” draft, for obvious reasons. Good writing is the product of revising and revising..and revising. It may take more time and effort than you realized, but it *is* a skill you can master.

### Highlight 11: “Who is the lead author, and who is going to oversee this project?” (Establish order of authorship and the process that all authors will follow.)

Before beginning the manuscript, the order of authors should be established. Especially important is identifying the lead author. Next, the group’s goals, each author’s contributions, processes for editing and reviewing, and deadlines should be clearly elucidated; it can be very helpful to express them in writing, and basic outlines have been published ( Primack, Gigliano, Parsons 2014). One suggestion for the process is for the lead author to serve as a “hub” who sends the manuscript to the other authors and then receives and incorporates their changes, rather than passing it around .. and around. By having all of these features in place *before* you launch the manuscript, you will obviate some problems that could arise during the course of writing the paper or at the time of submission. If problems should arise, you may want to consult some articles dealing with specific issues ( Albert & Wager 2003; Bennett *,* Gadlin, Levine-Finley 2010; Bennett and Gadlin 2012).

### Highlight 12: “Oh, no..the deadline was yesterday!” (Set and keep deadlines.)

Keeping deadlines is integral to the cooperative experience of writing with other individuals. Self-imposed deadlines are effective in improving task performance ( Ariely & Wertenbroch 2002). Regardless of the procedure your group decides to follow with regard to each author’s responsibility, deadlines for completion of work and collaborative discussion should be set and must be kept. One individual can hinder the successful production of a manuscript and cause undo stress and concern on the part of the other authors. You will want to make sure you are not that person!

## Conclusion

The medical educator has numerous resources for learning the “how-to” of writing the scholarly paper. However, successful writing requires far more than merely knowing the structure of the paper or how to get started. Indeed, many pitfalls await the unsuspecting writer. We present important ways to avoid becoming a victim of those pitfalls so that the writing experience can be less stressful and, hopefully, even enjoyable.

## Take Home Messages

• Know and adhere to publication ethics.
• Identify and state clearly the importance of your scholarly work (rhetorical statement).
• Set boundaries to protect your time and space.
• Be accountable to the other authors and keep deadlines.

## Notes On Contributors

B. Lee Ligon, PhD, MA, MAR, is department medical writer, editor, and educator, and a core faculty member of the Centers for Research, Innovation, and Scholarship in Medical Education, Department of Pediatrics, Baylor College of Medicine (BCM). She taught English literature and writing for more than 25 years at a private university, where she also created the Professional Writing Program.

Teri L. Turner, MD, MPH, MEd, is the Vice Chair of Education and Director for the Center for Research, Innovation and Scholarship in Medical Education, Department of Pediatrics, Baylor College of Medicine. She co-directs the Master Teacher Fellowship Program and is chair of the Academy of Distinguished Educators for BCM.

Satid Thammasitboon, MD, MHPE, is Associate Professor of Pediatrics, Critical Care at Baylor College of Medicine/Texas Children’s Hospital, and the Associate Director for the Center for Research, Innovation and Scholarship in Medical Education, Department of Pediatrics, Baylor College of Medicine.
