# Supplementary material for: Highlighting common pitfalls to avoid when writing the medical education manuscript
Source: MedEdPublish (2016). 2017 Jun 6;6:94. [Version 1] doi: 10.15694/mep.2017.000094 (PMC10885260; doi:10.15694/mep.2017.000094)
Supplement: Peer review history

# Reviewer report: Reviewer response for version 1

**DOI:** 10.21956/mep.19299.r28119

**Published:** 2017-06-29

**Authors:** Kanhua Yin

**Affiliations:** 1 Harvard University

This review has been migrated. The reviewer awarded 5 stars out of 5 Ligon and colleagues provided us with a great review of the key points, or the "pitfalls" in their words, in writing a medical education manuscript. It is a well-structured, reader-friendly, and, most importantly, insightful guideline. It is very practical and can be applied easily in the daily research works, especially for those who just start their explorations in medical education or other academic fields. I cannot agree with the authors more that premature submission never works and may even hurt someone’s academic career. Also, a good paper always need intensive revisions. Being patient and humble to the comments, suggestions or even criticism is a must-need for those who interested in achieving long-term success in any academic field. As a non-native English speaker, I would like to highlight the importance of language in writing. Although there are tons of professional language editing services in the academic fields, I still feel that someone should focus on improving their own language abilities rather than just rely on those services. Only practice makes perfect.Thank the authors again for their great paper.

Reviewer Expertise:

NA

No decision status is available

# Reviewer report: Reviewer response for version 1

**DOI:** 10.21956/mep.19299.r28120

**Published:** 2017-06-13

**Authors:** Subha Ramani

**Affiliations:** 1 Harvard Medical School, Brigham and Women's Hospital

This review has been migrated. The reviewer awarded 5 stars out of 5 I greatly enjoyed reading this paper on writing and will use these recommendations in my own writing. It is well written, well structured and well referenced. I agree with other reviewers that scholars beyond educators will find this paper useful. This is a different perspective on writing for scholarship which looks at important issues other than the actual writing. The authors have done a remarkable job of emphasizing areas such as ethics- authorship, plagiarism and citation; aims of discourse- the right audience, importance of the scholarly work and completeness; boundaries- making the time and creating the space to write; and accountability- time line, responsibilities of authors etc.I highly recommend this to all those interested in educational or other forms of scholarship.

Reviewer Expertise:

NA

No decision status is available

# Reviewer report: Reviewer response for version 1

**DOI:** 10.21956/mep.19299.r28122

**Published:** 2017-06-06

**Authors:** Trevor Gibbs

**Affiliations:** 1 AMEE

This review has been migrated. The reviewer awarded 5 stars out of 5 I very much enjoyed reading this article that looked at scholarship from a different and rarely used perspective. Although there was some overlap between the various sections, this was appropriate. I learned much from the article and I am glad to be shared with the very useful references. I think this paper will inform a useful discussion that we have with all of our junior faculty, new to academic writing and I hope will encourage others to add it to their workshops etc on academic writing and scholarship- well doneBTW, I don't think that there is a real word - mentoree- should it be mentee?

Reviewer Expertise:

NA

No decision status is available

# Reviewer report: Reviewer response for version 1

**DOI:** 10.21956/mep.19299.r28118

**Published:** 2017-06-06

**Authors:** Wolf Hautz

**Affiliations:** 1 Inselspital Bern

This review has been migrated. The reviewer awarded 4 stars out of 5 The authors provide a useful summary of tips and pitfalls commonly encountered in scholarly writing and I assume that many more experienced writers, reviewers and editors would agree.Although a number of articles on “How to get published” or “How to structure your article” have been previously published, the article by Lee et al. adds to them by focusing more on the process then on the content or structure of writing.On the one hand, a number of advices (such as following guidelines regarding authorship or reviewing the literature) are mere repetitions of what has been previously advocated. On the other, this repetition appears to be quite justified and necessary, which – in my opinion - shines some light on how well we as more experienced authors generally train our colleagues new to the field….An important issue touched upon in the article at some places is that of collaboration among the authors. Research in general and publishing specifically have become such complex tasks that approaching them as a team is almost imperative. Still, academic credit (such as awards, lead authorship, etc) is disproportionally awarded to individuals which clearly has the potential to undermine good team collaboration. The authors only superficially touch on the resulting problems and potential solutions. But maybe, this issue is worth a whole different paper.

Reviewer Expertise:

NA

No decision status is available

# Reviewer report: Reviewer response for version 1

**DOI:** 10.21956/mep.19299.r28121

**Published:** 2017-06-06

**Authors:** P Ravi Shankar

**Affiliations:** 1 American International Medical University

This review has been migrated. The reviewer awarded 4 stars out of 5 Thank you for the opportunity to review this interesting and well-written paper. The different online resources mentioned in highlight 4 are very useful. The service JANE has moved to http://jane.biosemantics.org/ Most of the authors’ descriptions will be useful to researchers in areas other than medical education also who are interested in writing up and submitting their work for publication. Many of these descriptions are available in the literature but the authors have brought these together in a single paper and have organized these into four categories. I enjoyed reading this paper and reflecting on the author’s suggestions. Medical researchers embarking on the process of scientific publication will find this to be of special interest.

Reviewer Expertise:

NA

No decision status is available
